# Age-related changes in microbial composition and function in cynomolgus macaques

**DOI:** 10.18632/aging.102541

**Published:** 2019-12-14

**Authors:** Jiajia Duan, Bangmin Yin, Wei Li, Tingjia Chai, Weiwei Liang, Yu Huang, Xunmin Tan, Peng Zheng, Jing Wu, Yifan Li, Yan Li, Wei Zhou, Peng Xie

**Affiliations:** 1 NHC Key Laboratory of Diagnosis and Treatment on Brain Functional Diseases, Chongqing Medical University, Chongqing, China; 2 The M.O.E. Key Laboratory of Laboratory Medical Diagnostics, The College of Laboratory Medicine, Chongqing Medical University, Chongqing, China; 3 Department of Neurology, The First Affiliated Hospital of Chongqing Medical University, Chongqing, China; 4 Department of Neurology, Army Medical Center of PLA, Chongqing, China

**Keywords:** age, gut microbiota, metagenomics, cynomolgus macaques

## Abstract

Age can significantly affect human physiology and disease risk. Recent studies have shown that age may affect the composition and function of the gut microbiota, but the underlying mechanisms remain largely unknown. Non-human primates are an ideal model for uncovering how age shapes the gut microbiota, as their microbial composition is highly similar to that of humans and is not easily affected by confounding factors. Here, using the 16S rRNA and metagenomic sequencing methods, we characterized the microbial phenotypes of 16 female cynomolgus macaques from three age groups (young, adult and old). Our findings revealed significant differences in microbial composition among the three groups. With increased age, the relative abundances of *Veillonellaceae*, *Coriobacteriaceae* and *Succinivibrionaceae* were significantly increased, *Ruminococcaceae* and *Rikenellaceae* were significantly decreased at the family level. Functional enrichment showed that genes that differed among the three groups were mainly involved in arginine biosynthesis, purine metabolism and microbial polysaccharides metabolism. Moreover, CAZymes corresponding to polysaccharide degrading activities were also observed among the three groups. In conclusion, we characterized the composition and function of the gut microbiota at different ages, and our findings provide a new entry point for understanding the effects of age on the human body.

## INTRODUCTION

Aging is an inevitable phenomenon in both humans and animals. In three key phases of the life cycle, namely young, adult and old, individuals present distinct biological characteristics and disease risks [1]. Thus, the identification of biological characteristics in particular age phases is valuable to understanding the development of diseases, thereby aiding strategies to prevent disease and prolong lifespan.

The gut microbiota is the largest flora that is most directly linked to the external environment in both humans and animals. The composition and function of the gut microbiota plays a vital role in maintaining the host’s health. Emerging research has reported that some microbial signatures are related to age. For example, during the neonatal period, Firmicutes acts as the dominant flora, whereas the abundance of Proteobacteria and Actinobacteria starts to increase from 3 to 6 months [2]. With the cessation of breast milk, the increased abundance of Firmicutes represents gradual maturation of the gut microbiota [3]. The dynamic changes in the microbial composition tend to be stable by 3 years, which is highly similar to that in adults [4]. In adults, the microbial composition predominantly comprises the phyla Bacteroidetes and Firmicutes [5]. By contrast, the microbial composition in older individuals displays a higher Bacteroidetes ratio compared with their younger counterparts [6]. Evidence has been reported that the microbial composition may affect the rate of aging [7, 8]. Moreover, changes in gut physiology are modulated by age, such as degenerative changes in the enteric nervous system and gastric dysmotility, which have profound effects on the composition, diversity and functional characteristics of the gut microbiota [9]. These findings in humans and rodents provide valuable clues for further research. However, given that microbial composition is vulnerable to various confounding factors, such as social status, lifestyle, dietary and genetic diversity, potential bias cannot be completely ruled out. Moreover, using the 16S ribosomal RNA (rRNA) sequencing method, previous studies focused on changes in microbial composition. Further studies using shotgun metagenomics methods are required to identify the functional activity linked with age.

Gut microbiota of the non-human primates, cynomolgus macaques, is more similar to that of humans than that of rodents [10], and is less affected by environmental and lifestyle factors when an identical diet and environmental condition are provided. Moreover, it has been reported that cynomolgus macaques can serve as a useful model for simulating human aging [11]. Here, to investigate the key age-related microbes and microbial functions, we selected 16 cynomolgus macaques aged from 2 to 20 years, which showed high homogeneity in their living environments, daily diet and health condition. Using a combination of 16S rRNA and shotgun metagenomic sequencing methods, we sought to compare microbial composition and function for three different age phases (young, adult and old).

## RESULTS

### General characteristics of the recruited monkeys

Sixteen healthy monkeys were included in this study under the same feeding and living conditions. According to age, these monkeys were divided into three groups: young, adult and old. The detailed characteristics of the recruited monkeys are shown in Supplementary Table 1.

### Similar within-sample microbial diversity among the three groups

**Figure 1:**
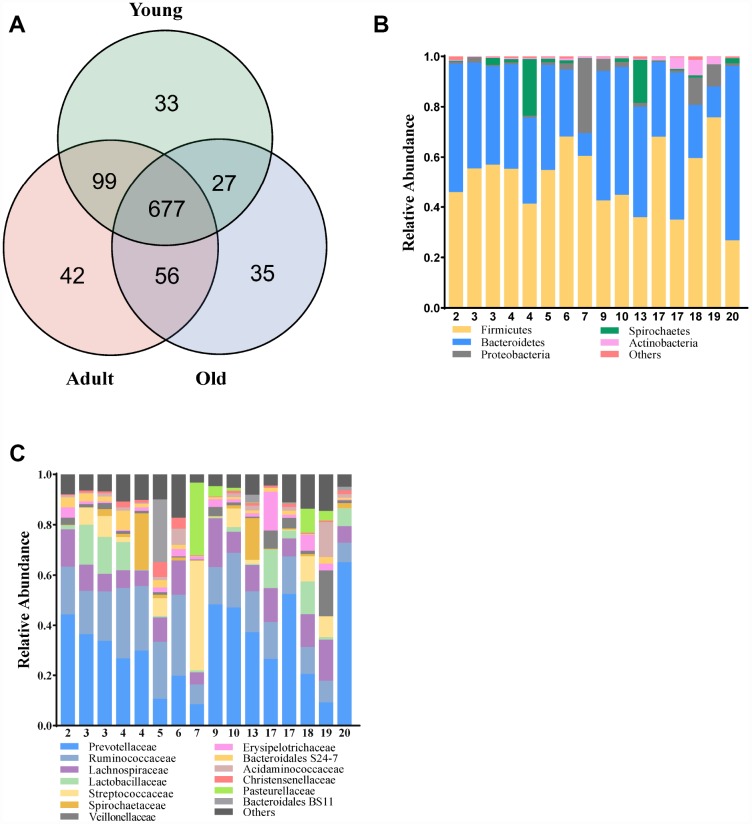
**Comparison of the microbial composition between the three groups.** (**A**) Venn diagram depicting OTU richness and the overlap in microbial communities between the young (green), adult (red) and old (blue) monkeys. (**B**, **C**) Relative abundance of OTUs assigned at the phylum and family levels.

To characterize the gut composition of monkeys among the three age groups, 16S rRNA gene sequencing was initially used. In total, we identified 905,921 high-quality reads, ranging from 47,691 to 66,725 per sample, with an average read length of 439.06 bp (439.06 ± 2.88 bp). A Venn diagram showed that three age groups shared 677 of the 969 operational taxonomic units (OTUs, defined based on 97% sequence similarity), whereas 33, 42 and 35 OTUs were unique to young, adult and old monkeys, respectively (Figure 1A). At the phylum and family levels, the relative abundance of microbes in the three groups is presented in Figure 1B, 1C. Our findings revealed that the microbial composition of cynomolgus monkeys was dynamically changed with age. The phyla Firmicutes, Bacteroidetes and Proteobacteria dominated in the gut microbiota of monkeys. *Prevotellaceae* and *Ruminococcaceae* represented the most abundant families among the three groups, but showed a downward trend with age.

The α-diversity, including microbial community richness (Chao, Ace) and diversity (Shannon, Invsimpson), was compared among the young, adult and old groups. A downward trend was observed in these indexes with increased age, although no statistical difference was detected (Wilcoxon rank-sum test, all p values>0.05) (Supplementary Figure 1A–1D).

### Significant differences in the microbial composition among the three groups

**Figure 2:**
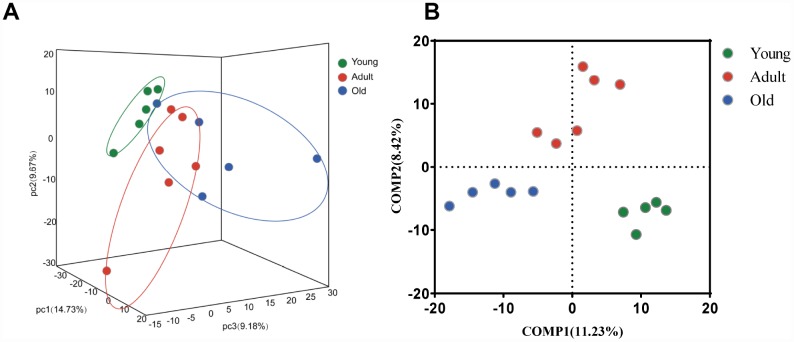
**Comparison of the microbial composition between the young, adult and old groups.** (**A**) 3-D Principle component analysis (PCA) plot of samples along principle component (PC) 1,2 and 3, which explained 14.73%, 9.67% and 9.18% of the total variance, respectively. (**B**) Partial least squares discriminant analysis (PLS-DA) plot of gut microbiota among three groups: young (n=5, 2–4 years, green dots), adult (n=6, 5–13 years, red dots) and old (n=5, 17–20 years, blue dots).

To further explore whether the microbial composition of the young, adult and old groups differed significantly, β-diversity analysis was carried out. Firstly, at the OTU level, a 3-D principal component analysis (PCA) plot displayed that there was a discriminative trend among the three groups, but no statistical difference was detected (PERMANOVA, all p values>0.05) (Figure 2A). Interestingly, we found that samples from the adult group were distributed in the central region, whereas samples from the young and old groups were distributed on both sides. This finding suggested that gut microbial composition may change dynamically with age. Moreover, we found that the gut microbiota of the adult group showed more variation than the young group, which was consistent with previous reports in humans [12]. To further display the differences of the bacterial communities among the three groups, the partial least squares discriminant analysis (PLS-DA) was performed, the bacterial communities of the three groups clustered separately (Figure 2B), indicating distinctive fecal microbial communities among three groups.

**Figure 3:**
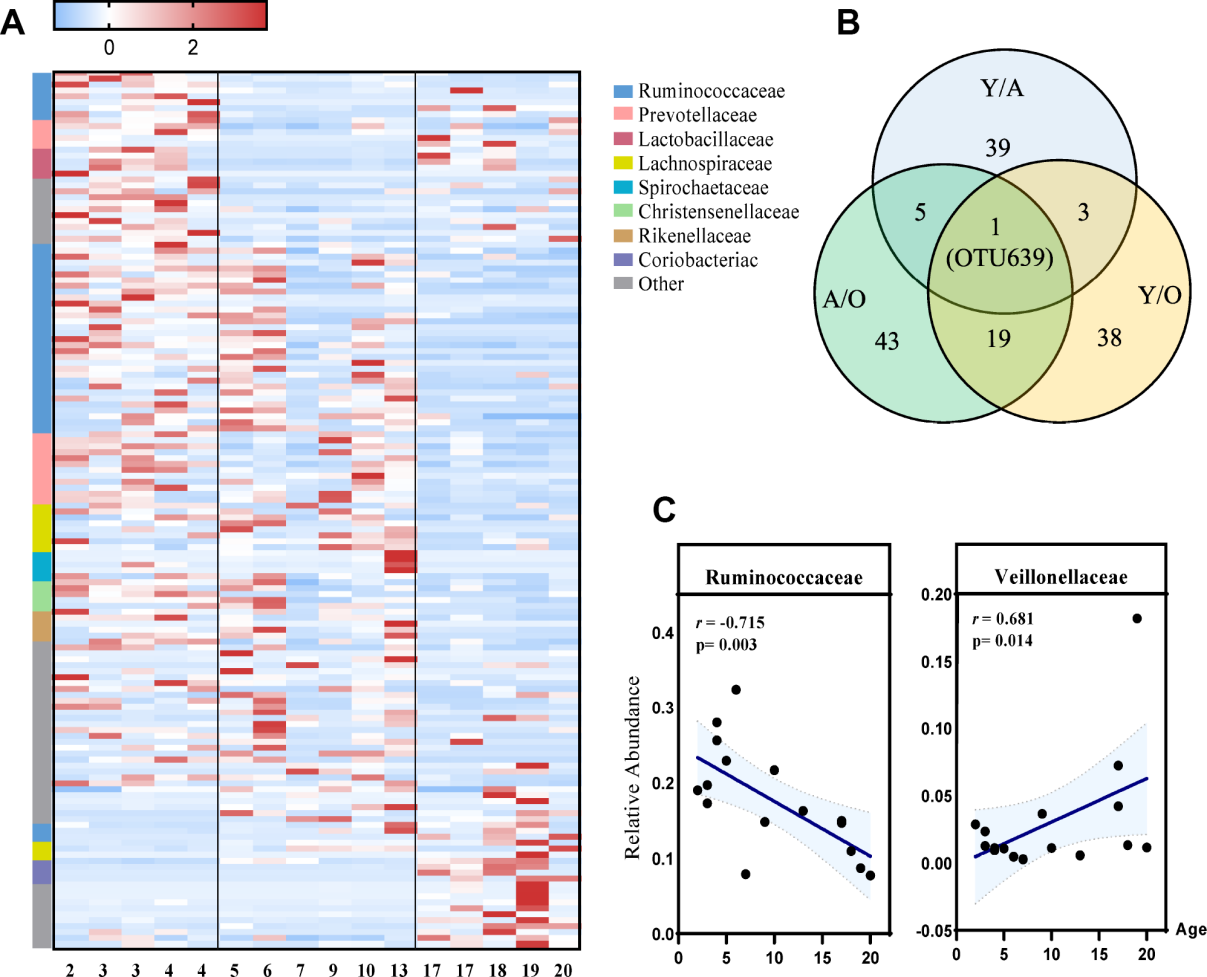
**The most differentially expressed taxa among the three groups.** (**A**) Heatmap of the 148 discriminative OTU abundances among the young, adult and old groups (LDA>2.0). OTUs (raw) were sorted by taxa and enriched groups, samples (column) were sorted by age. The intensity of color (blue to red) indicated the score normalized abundance for each OTU. (**B**) Venn diagram for different OTUs among the three groups. Blue designates enriched taxa between the young and adult groups; green designates enriched taxa between the adult and old groups; yellow designates the enriched taxa between the young and old groups. (**C**) Scatter diagram of the relative abundances of the age-related microbial families *Ruminococcaceae* and *Veillonellaceae*. The correlation was tested by Pearson’s correlation analysis and was adjusted by partial correlation analysis.

To identify the profiles of gut microbiota at different age phases, LEfSe analysis was performed. This analysis identified 148 different OTUs responsible for this discrimination (Figure 3A, Supplementary Figure 2A). With increased age, the relative abundances of *Veillonellaceae* (p=0.014), *Coriobacteriaceae* (p=0.022) and *Succinivibrionaceae* (p=0.009) were significantly increased, whereas *Ruminococcaceae* (p=0.003) and *Rikenellaceae* (p=0.009) were significantly decreased with age at the family level (Figure 3C, Supplementary Figure 3). Moreover, among these differentially represented microbes, we found that *Ruminiclostridium 9* was the only OTU that dynamically changed in all three groups (Figure 3B).

### Alternations of arginine and purine metabolism as well as changes in microbial peptidoglycan with age

**Figure 4:**
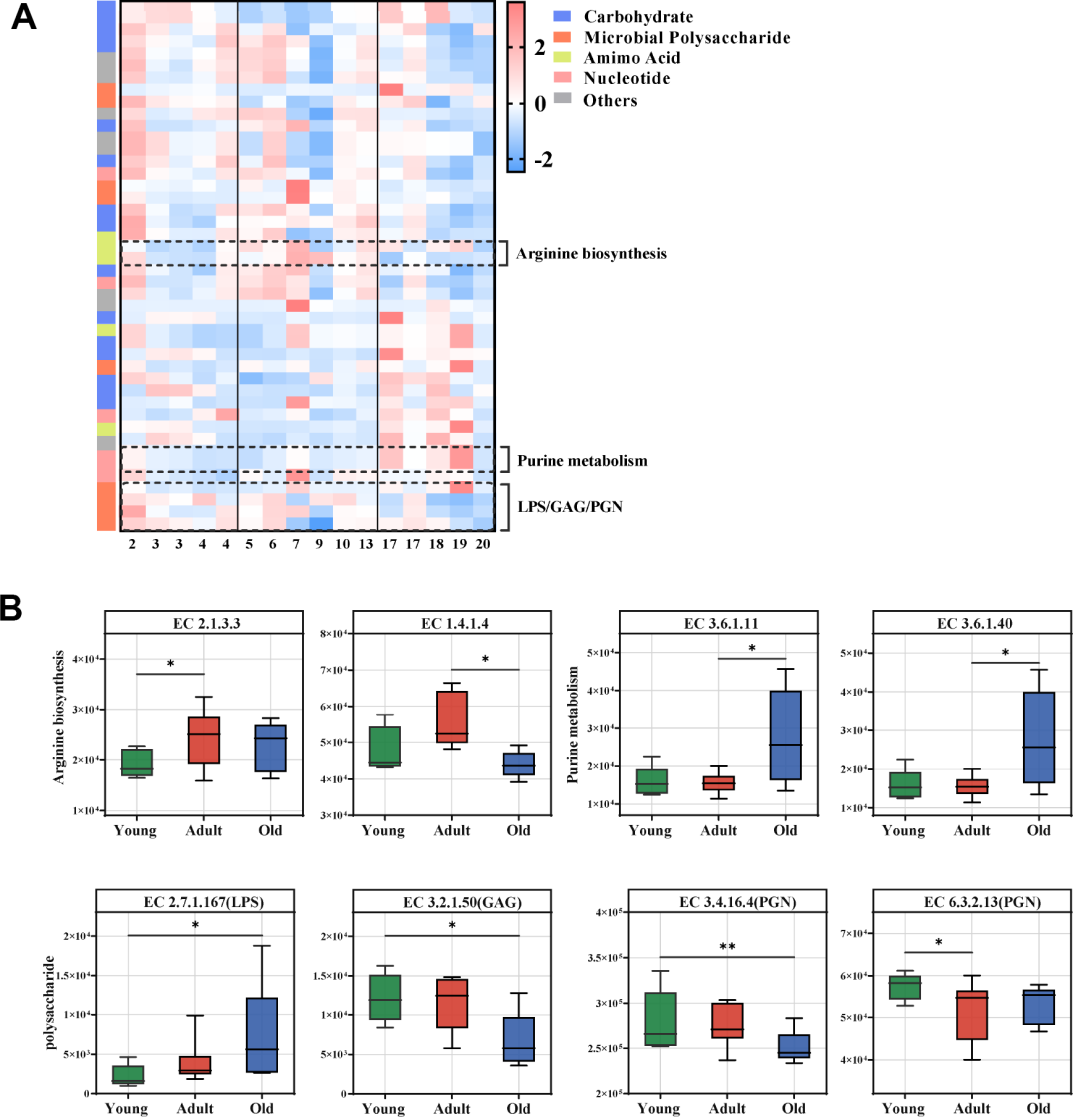
**Age-related microbial functions in the KEGG pathway.** Different KEGG enzymes identified by LEfSe analysis of the metagenomic sequences (LDA>2.0). (**A**) Heatmap of the abundances of different enzymes. Enzymes (raw) were sorted by taxa and enriched group, and samples (column) were sorted by age. The intensity of the color (blue to red) indicates the score normalized abundance for each enzyme. (**B**) Boxplot for the marked KEGG enzymes in different age groups. ECs were classified into pathways for arginine, purine and microbial polysaccharide metabolism. The abundances of different ECs were calculated by reads number. LEfSe was used to detect features with significantly different abundances using the Kruskal–Wallis rank sum test, and LDA was performed to evaluate the effect size of each feature. *P<0.05 **P<0.01. LPS, lipopolysaccharide; GAG, glycosaminoglycan; PGN, peptidoglycan.

To characterize the functions encoded by the gut microbial DNA, we performed whole-genome shotgun sequencing of stool samples obtained from the three groups. A total of 1,602,244,870 filtered reads and 45,088,293 open reading frames (ORFs) were used for functional annotation in the KEGG and CAZy databases. Using linear discriminant analysis (LDA) effect size (LEfSe) analysis, we identified 44 KEGG enzymes responsible for discrimination among the three groups (LDA>2.0). Pathway-enrichment analysis revealed that these differential enzymes were mainly involved in amino acid metabolism (especially arginine metabolism) and nucleotide metabolism (purine metabolism), as well as microbial polysaccharides including lipopolysaccharide (LPS), glycosaminoglycan (GAG) and peptidoglycan (PGN) (Figure 4A, Supplementary Figure 2B). The marked enzymes involved in arginine and purine metabolism, as well as microbial polysaccharide are presented in Figure 4B. Our data showed that arginine biosynthesis and purine metabolism were enriched in the adult and old groups, respectively. For polysaccharide metabolism, GAG and PGN were significantly enriched in the young group, whereas LPS was enhanced in the old group.

### Age-related microbial functions in CAZymes

**Figure 5:**
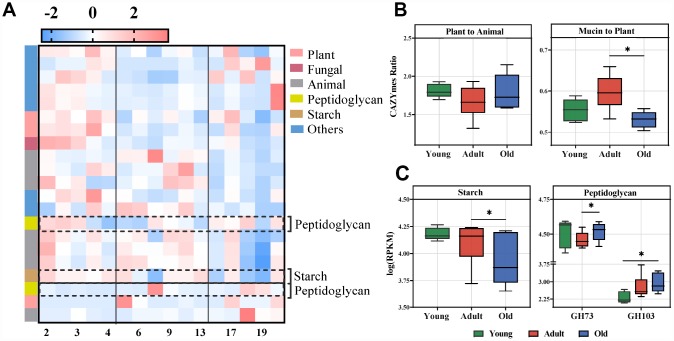
**Age-related microbial functions in CAZymes and food utilization.** Different CAZymes identified using LEfSe analysis of the metagenomic sequences (LDA>2.0). (**A**) Heatmap of the abundances of different CAZymes. CAZymes (raw) were sorted by taxa and enriched group, and samples (column) were sorted by age. The intensity of color (blue to red) indicates the score normalized abundance for each enzyme. (**B**) The ratio of CAZymes represented within the metagenomes related to plant and animal carbohydrate utilization (left) or the ratio of mucin glycan to plant carbohydrate utilization (right) in the cynomolgus macaques. The boxplot distributions were tested using the nonparametric two-sided Wilcoxon rank sum test. (**C**) Boxplot for the marked CAZymes in different age groups. Representative CAZymes were classified into pathways for starch (left) and microbial peptidoglycan (right). The abundances of different CAZymes were calculated by the log RPKM. LEfSe detected the features with significantly different abundances using the Kruskal–Wallis rank sum test, and LDA was performed to evaluate the effect size of each feature. *P<0.05, **P<0.01.

To further uncover the function of the gut microbiota, the differential CAZymes among the three groups were analyzed. In total, we identified 21 CAZymes responsible for the discrimination among the three groups (LDA>2.0) (Figure 5A, Supplementary Figure 2C). These CAZymes were mainly involved in the utilization of plant and animal carbohydrates, and microbial polysaccharide metabolism (Figure 5A, Supplementary Figure 4). The gut microbiota of the adult group had a greater utilization capacity for mucin rather than plants compared with the old group (Figure 5B). Interestingly, we found that the CAZymes related to PGN were also significantly changed (Figure 5C), which was consistent with the altered KEGG pathways. Moreover, analysis of CAZyme profiles showed that the microbiota of the adult group had greater functional capacity for the utilization of starch than the old group, and PGN utilization occurred predominantly in the old group.

## DISCUSSION

The gut microbiota coexists with the human body and changes with age. In this study, we explored age-related changes in microbial composition and function in three representative age phases in monkeys. We found that the microbial composition of monkeys differed significantly at different age phases. A panel of microbes such as *Veillonellaceae*, *Coriobacteriaceae*, *Ruminococcaceae* and *Rikenellaceae* dynamically changed in prevalence with increased age. Functionally, we found that arginine biosynthesis, purine metabolism and microbial polysaccharides metabolism were dynamically changed. Our findings provide new insight into how age influences the host through the gut microbiota.

Previous clinical and rodent studies have suggested that age may affect the composition of the gut microbiota [4, 13, 14]. Here, monkeys were used to investigate this issue and this animal model has the following advantages: (i) the microbial composition of monkeys is highly similar to that of humans [10], which makes it easier to translate these findings into human research; (ii) it can effectively avoid the influences of confounding factors such as living environment and genetic background; (iii) nonhuman primates exhibit similar key life span metrics as humans [11]. Here, we characterized the composition and function of the gut microbiota at three representative age phases, which is a new development in this field.

Our results showed that the α-diversity of the gut microbiota in cynomolgus macaques was reduced with age, which was consistent with previous human studies [15]. Moreover, we found that the microbial composition of the three groups was significantly different. Firmicutes and Bacteroidetes were the dominant phyla in both humans and cynomolgus macaques [16, 17]. Similar to human studies, we found that, compared with the young and adult groups, the old group showed a slight increase in Firmicutes, whereas Bacteroidetes gradually decreased after youth [18]. We also found some differences between monkey and human studies, for example, Actinobacteria was increased in prevalence with age in monkeys, whereas human studies showed the opposite trend. This may result from the inevitable confounding factors. With increased age, the relative abundances of *Veillonellaceae* and *Coriobacteriaceae* were significantly increased, and *Ruminococcaceae* and *Rikenellaceae* were significantly decreased at the family level. There is evidence to confirm that the family *Veillonellaceae* is associated with age-related diseases such as atherosclerosis and stroke [19]. Another family *Ruminococcaceae* plays a vital role in the maintenance of gut health through degrading cellulose and hemicellulose components of plant material by CAZymes and transporters. These compounds are fermented and converted into short-chain fatty acids (mainly acetate, butyrate and propionate), which are absorbed by the host and are important for metabolic and immunological homeostasis [20]. Our finding showed that the relative abundance of *Ruminococcaceae* was negatively correlated with age. Consistent with our findings, previous studies showed that *Ruminococcaceae*, one of the core microbiota, becomes less abundant in older people, whereas some taxa associated with unhealthy aging emerge. These findings suggested that *Ruminococcaceae* may have a positive effect on the aging process [21, 22]. Interestingly, at the species level, we found that *Ruminiclostridium_9* shared across the three age groups. Previous research showed that it played a role in controlling obesity development [23]. Further studies are required to determinate whether supplementation of this strain can result in body fat reduction. Here, we also found that some microbes belonging to *Lachnospiraceae* and *Prevotellaceae* dynamically changed with age, which needs to be addressed in further investigations.

At the functional level, we found that arginine biosynthesis, purine metabolism and microbial polysaccharide metabolism were dynamically changed with age. With increased age, arginine biosynthesis was significantly upregulated in the adult group. As arginine can improve immune function and overall health, compelling evidence shows that enteral or parenteral administration of arginine reverses endothelial dysfunction associated with major cardiovascular risk factors, including aging [24]. This finding suggests that the body may regulate self-protection with age. Moreover, purine metabolism was increased in the old group. Uric acid, a metabolite of purine, mainly exerts antioxidant activity [25], suggesting increased oxidative stress in the older population. Consistent with this speculation, we also observed increased biosynthesis of LPS in the old group. LPS plays a key role during host–pathogen interactions and chronic inflammation [26]. We also found that the gut microbiota of the young group was enriched in genes involved in the degradation of glycosaminoglycan, which has been linked with skeletal growth and animal development [27].

Additionally, we observed a higher relative abundance of enzymes involved in PGN biosynthesis in both the young and adult age groups relative to the old age group. In agreement with these findings, the CAZymes related to PGN catabolism were also significantly increased in the old group relative to the young or adult groups. A recent study showed that PGN can cross the blood–brain barrier, and influence brain molecules and functions through activating the PGN-pattern recognition receptor (PGN-PRR) pathway in an age-specific manner [28]. This finding facilitates our understanding of age-related brain changes or diseases.

It should be noted that our research had the following limitations. Firstly, our study was based on a cross-sectional design. The longitudinal collection of feces at different age stages would be the best way to study the effects of age on the gut microbiota, but the dynamic collection of feces over a decade time-span is challenging, and the gut microbiota may be significantly changed under long-term storage conditions. In addition, consistent with other monkey research, the sample size was relatively limited. Thus, our findings may require further validation. Finally, we provided evidence of alternations in age-related microbial structure and function, but further in-depth research is required to confirm this.

In conclusion, we provide initial evidence as to how age shapes the structure and function of the gut microbiota based on monkey studies. Our findings provide insight into how the gut microbiota physiologically affects the host at different age stages.

## MATERIALS AND METHODS

### Site and sample collection

Our study was conducted in Zhongke Experimental Animal Co., Ltd. (hereafter referred to as "Zhongke"), which is a breeding base for cynomolgus monkeys in Suzhou, China (E 31°07'03" to 31°07'06", N 120°19'08" to 120°19'15"). The housing conditions and animal care procedures were detailed in a previous report [29] and were in accordance with Chinese regulatory requirements and accredited by the Association for the Assessment and Accreditation of Laboratory Animal Care International (AAALAC). In brief, monkeys in Zhongke were provided standard sanitation, an adequate and regular diet, and a stable social structure. All procedures involving non-human primates were approved by the Animal Care and Use Committee of Chongqing Medical University and were in compliance with the Guide for the Care and Use of Laboratory Animals [29].

All 16 cynomolgus macaques, as listed in supplemental data, were randomly selected from different enclosures. In accordance with previous reports [30], the 16 macaques were assigned into three age groups: young (2–4 years), adult (5–15 years) and old (17–20 years). All macaques were housed in free enclosures without manual intervention measuring 8 × 3 × 3 m (L × W × H), were provided with water ad libitum, and were fed daily with fresh fruit, vegetables and compound high-nutrition monkey food [29]. Collection of fecal samples occurred immediately after the first defecation using a germ-free device, and samples were stored in liquid nitrogen before being transported on ice to maintain a temperature chain of < −80°C.

### 16S rRNA gene sequencing and data processing

DNA extraction and amplification of the 16S rRNA gene were performed as previously described [31]. Briefly, DNA was extracted from stool samples using the E.Z.N.A® DNA kit (Omega Bio-Tek, USA). The V3-V4 hypervariable regions of the bacterial 16S rRNA gene were amplified with primers 338F (5ʹ- ACTCCTACGGG AGGCAGCAG-3ʹ) and 806R (5ʹ-GGACTACHVGGG TWTCTAAT-3ʹ) by the thermocycler PCR system (GeneAmp 9700, ABI, USA). The PCR conditions were 3 min of denaturation at 95°C, followed by 27 cycles of 30 s at 95°C for denaturation, 30 s for annealing at 55°C, and 45 s for elongation at 72°C, and a final extension at 72°C for 10 min. The PCR was performed in triplicate in a 20 μL mixture containing 4 μL of 5 × FastPfu buffer, 2 μL of 2.5 mM dNTPs, 0.8 μL of each primer (5 μM), 0.4 μL of FastPfu polymerase and 10 ng of template DNA. The resulting PCR products were separated on 2% agarose gels, purified using the AxyPrep DNA Gel Extraction Kit (Axygen Biosciences, Union City, CA, USA) and quantified using QuantiFluor™-ST (Promega, USA) according to the manufacturer’s protocol. Purified amplicons were pooled in equimolar concentrations and paired-end sequenced (2 × 300) on an Illumina MiSeq platform (Illumina, San Diego, USA) according to the standard protocols by Majorbio Bio-Pharm Technology Co. Ltd. (Shanghai, China). The raw reads were deposited into the NCBI Sequence Read Archive (SRA) database (Accession Number: SRP218284).

Raw fastq files were demultiplexed and quality-filtered using QIIME (version 1.9.1, http://qiime.org/). The 250 bp reads were truncated at any site of more than three sequential bases receiving an average quality score of <20. Low-quality reads (reads shorter than 50 bp or barcode/primer errors or reads with a quality value <20) were removed.

Operational taxonomic units (OTUs) were clustered with 97% similarity cutoffs using UPARSE (version 7.1, http://drive5.com/uparse/) with a novel ‘greedy’ algorithm that performs chimera filtering and OTU clustering simultaneously. The taxonomy of each 16S rRNA gene sequence was analyzed by the RDP classifier algorithm (http://rdp.cme.msu.edu/) against the Silva (SSU128) 16S rRNA database using a confidence threshold of 70%.

### Shotgun metagenome sequencing and data processing

The fecal samples were further investigated by metagenomic sequencing. The concentration of the extracted DNA was determined by the TBS-380 method, its purity was measured with a NanoDrop 2000 spectrophotometer, and its quality was confirmed by electrophoresis on a 1% agarose gel. The DNA was then fragmented to an average size of about 300 bp using Covaris M220 (Gene Company Limited, China) for paired-end library construction. A paired-end library was constructed using the TruSeq™ DNA Sample Prep Kit (Illumina, San Diego, CA, USA). Adapters containing the full complement of sequencing primer hybridization sites were ligated to the blunt-end of fragments. Paired-end sequencing was performed on the Illumina HiSeq4000 platform (Illumina Inc., San Diego, CA, USA) at Majorbio Bio-Pharm Technology Co., Ltd. (Shanghai, China) using the HiSeq 3000/4000 PE Cluster Kit and the HiSeq 3000/4000 SBS Kit according to the manufacturer’s instructions (www.illumina.com). Sequence data associated with this project have been deposited in the NCBI Short Read Archive database (Accession Number: SRP218382).

Adapter sequences were stripped from the 3' and 5' ends of the paired end Illumina reads using SeqPrep (https://github.com/jstjohn/SeqPrep). Sickle (https://github.com/najoshi/sickle) was used to remove the low-quality reads (length <50 bp or with a quality value <20 or having N bases).

The metagenomics data obtained were assembled using MEGAHIT [32] (https://github.com/voutcn/megahit). The final assembly containing contigs of 300 bp or more, was used for further gene prediction and annotation.

The prediction of ORFs from each assembled contig was performed using MetaGene software [33] (http://metagene.cb.k.u-tokyo.ac.jp/). The predicted ORFs with lengths of 100 bp or over were retrieved and translated into amino acid sequences.

All genes predicted to have 95% sequence identity (90% coverage) were clustered using CD-HIT software [34] (http://www.bioinformatics.org/cd-hit/), and representative sequences containing the longest sequences from each cluster were used to construct non-redundant gene catalogs. High quality reads were mapped to the representative sequences with 95% identity using SOAPaligner [35] (http://soap.genomics.org.cn/) to evaluate gene abundance in each sample.

KEGG annotation was conducted using BLASTP (Version 2.2.28+) against the Kyoto Encyclopedia of Genes and Genomes database [36] (http://www. genome.jp/keeg/) with an e-value cutoff of 1e-5. A total of 362,971,704 enzymes were annotated by the KEGG database. Carbohydrate-active enzyme annotation was conducted using hmmscan (http://hmmer.janelia.org/search/hmmscan) against the CAZy database version 5.0 (http://www.cazy.org/) with an e-value cutoff of 1e-5. A total of 60,024,802 enzymes were annotated by the CAZy database.

### Statistical analysis

The α-diversity indexes were assessed according to species richness (Ace and Chao), species evenness (Shannon) and species diversity (Simpson). Beta diversity was assessed with package ‘vegan’ in R (version R-3.3.1), and generated on the basis of principal component analysis (PCA). Descriptive modelling and discriminative variable selection were evaluated by partial least squares-discriminant analysis (PLS-DA). PERMANOVA was performed to identify differences in β-diversity among three groups. The key bacterial taxa, and the CAZymes and KEGG categories responsible for discrimination among the groups, were identified using linear discriminant analysis effective size (LEfSe; http://huttenhower.sph.harvard.edu/galaxy/root?tool_id=lefse_upload) [37]. Only LDA values >2.0 at a P value <0.05 were considered significantly enriched.

Statistical analyses were conducted using the software SPSS, R package, and plots were generated from R and GraphPad Prism version 8.0. The strategy of multiple cooperation was one-against-all when performing LEfSe. One-way ANOVA or the Kruskal–Wallis H test were performed to assess the alpha diversity and abundance of some species among the three age groups, and the two-tailed Student’s t-test or Wilcoxon rank-sum test were performed to determine differences between each set of two groups. The correlation between microbial abundance and age was tested by Pearson correlation analyses and adjusted by partial correlation analysis to exclude the confounders for controlling the false discovery rate (FDR). Adjustments were performed with SPSS (version 22.0).

## Supplementary Material

Supplementary Figures

Supplementary Table 1
