# Supplementary material for: Age-related changes in microbial composition and function in cynomolgus macaques
Source: Aging (Albany NY). 2019 Dec 14;11(24):12080–96. doi: 10.18632/aging.102541 (PMC6949106; doi:10.18632/aging.102541)
Supplement: Supplementary Figures [file aging-11-102541-s002..pdf]

SUPPLEMENTARY FIGURES

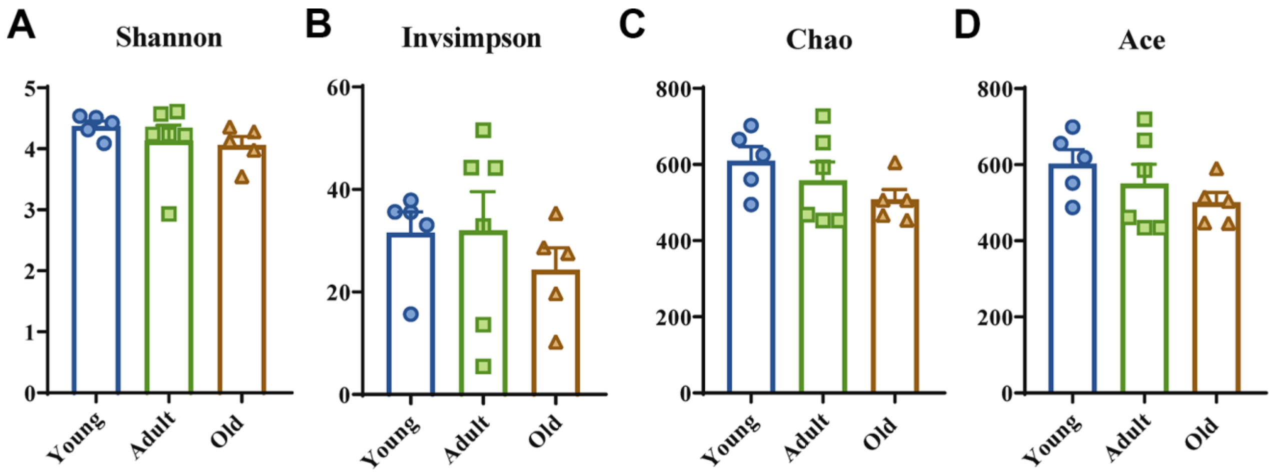

Supplementary Figure 1. Gut microbial characteristics of monkeys at the α- diversity analysis. Comparing the microbial composition of three groups based on α-diversity analysis. (**A**–**D**) In these four indexes (Shannon, Invsimpson, Chao and Ace), there is no significant difference among the three groups (Young, n=5; Adult, n=6; Old, n=5).

A

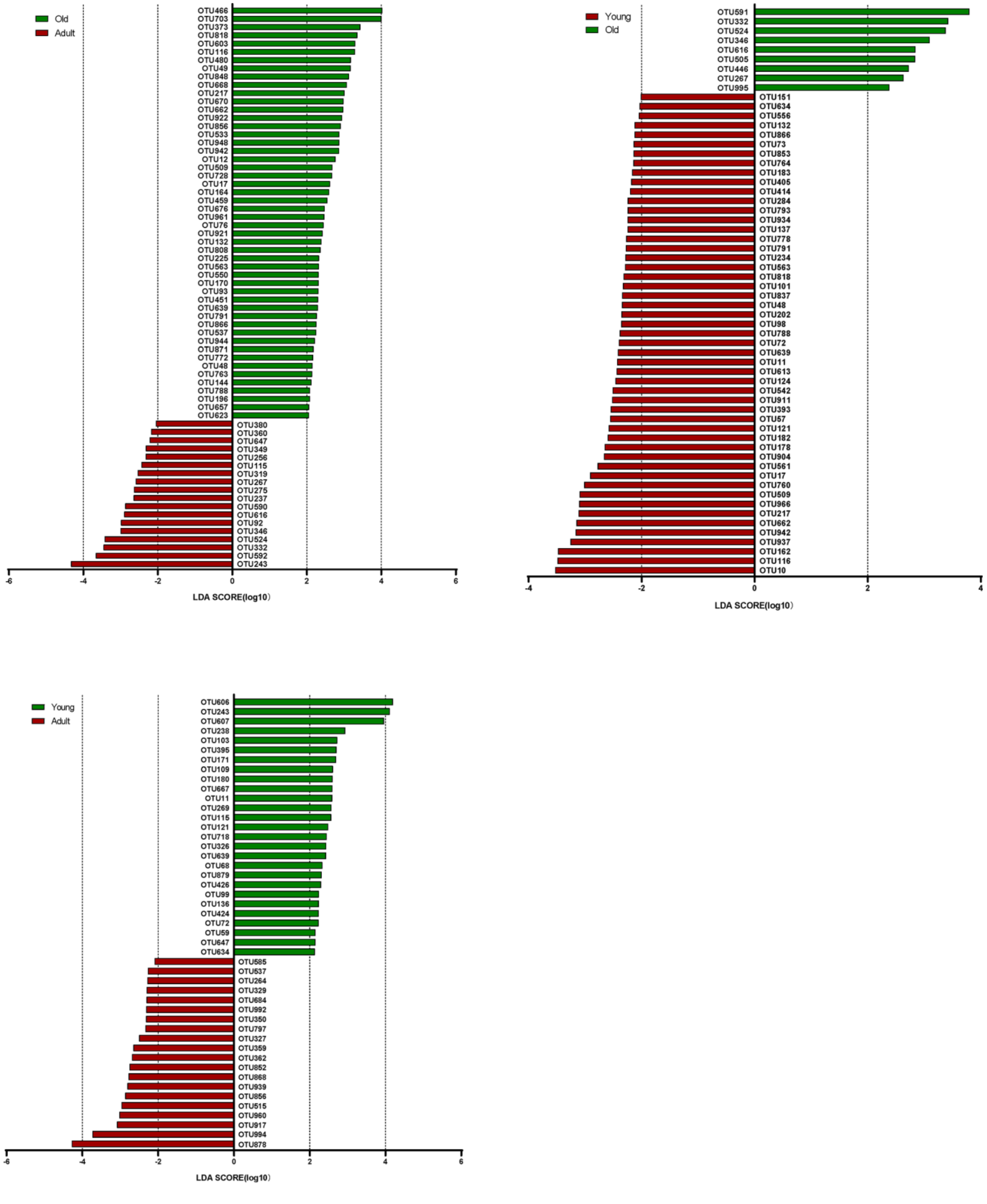
Old
Adult
OTU466
OTU703
OTU373
OTU818
OTU603
OTU116
OTU480
OTU49
OTU848
OTU668
OTU217
OTU670
OTU662
OTU922
OTU856
OTU533
OTU948
OTU942
OTU12
OTU509
OTU728
OTU17
OTU164
OTU459
OTU676
OTU961
OTU76
OTU921
OTU132
OTU808
OTU225
OTU563
OTU550
OTU170
OTU93
OTU451
OTU639
OTU791
OTU866
OTU537
OTU944
OTU871
OTU772
OTU48
OTU763
OTU144
OTU788
OTU196
OTU657
OTU623
OTU380
OTU360
OTU647
OTU349
OTU256
OTU115
OTU319
OTU267
OTU275
OTU237
OTU590
OTU616
OTU92
OTU346
OTU524
OTU332
OTU592
OTU243
-6
-4
-2
0
2
4
6
LDA SCORE(log10)
Young
Old
OTU591
OTU332
OTU524
OTU346
OTU616
OTU505
OTU446
OTU267
OTU995
OTU151
OTU634
OTU556
OTU132
OTU866
OTU73
OTU853
OTU764
OTU183
OTU405
OTU414
OTU284
OTU793
OTU934
OTU137
OTU778
OTU791
OTU234
OTU563
OTU818
OTU101
OTU837
OTU48
OTU202
OTU98
OTU788
OTU72
OTU639
OTU11
OTU613
OTU124
OTU542
OTU911
OTU393
OTU57
OTU121
OTU182
OTU178
OTU904
OTU561
OTU17
OTU760
OTU509
OTU966
OTU217
OTU662
OTU942
OTU937
OTU162
OTU116
OTU10
-4
-2
0
2
4
LDA SCORE(log10)
Young
Adult
OTU606
OTU243
OTU607
OTU238
OTU103
OTU395
OTU171
OTU109
OTU180
OTU667
OTU11
OTU269
OTU115
OTU121
OTU718
OTU326
OTU639
OTU68
OTU879
OTU426
OTU99
OTU136
OTU424
OTU72
OTU59
OTU647
OTU634
OTU585
OTU537
OTU264
OTU329
OTU684
OTU992
OTU350
OTU797
OTU327
OTU359
OTU362
OTU852
OTU868
OTU939
OTU856
OTU515
OTU960
OTU917
OTU994
OTU878
-6
-4
-2
0
2
4
6
LDA SCORE(log10)

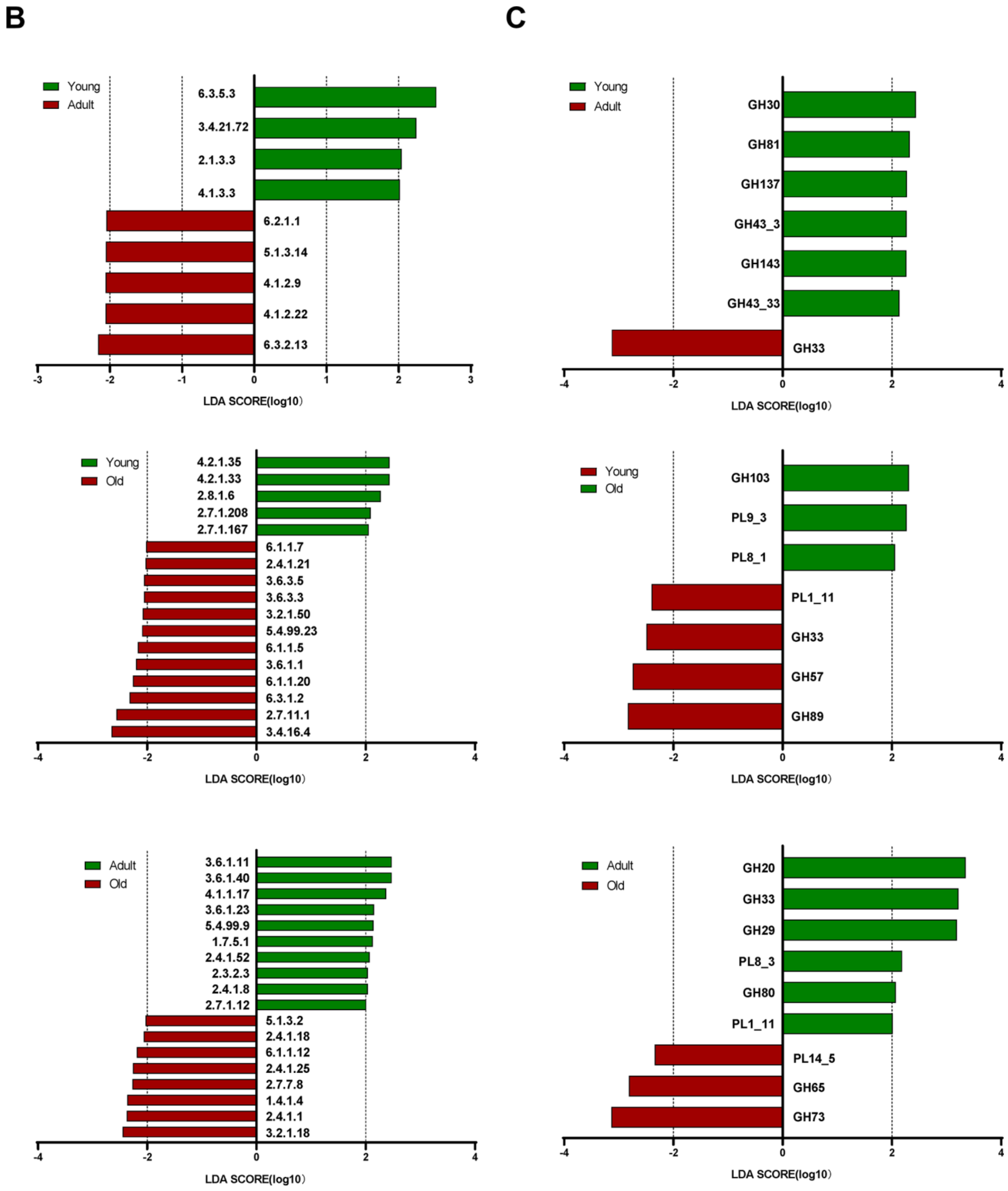

Supplementary Figure 2. The OTU and microbial functions differentially represented among three groups. OUT (**A**), KEGG enzymes (**B**) and CAZymes (**C**) differentially represented among the three age groups identified by Linear discriminant analysis (LDA) Effect Size (LEfSe) (LDA>2.0,p<0.05).

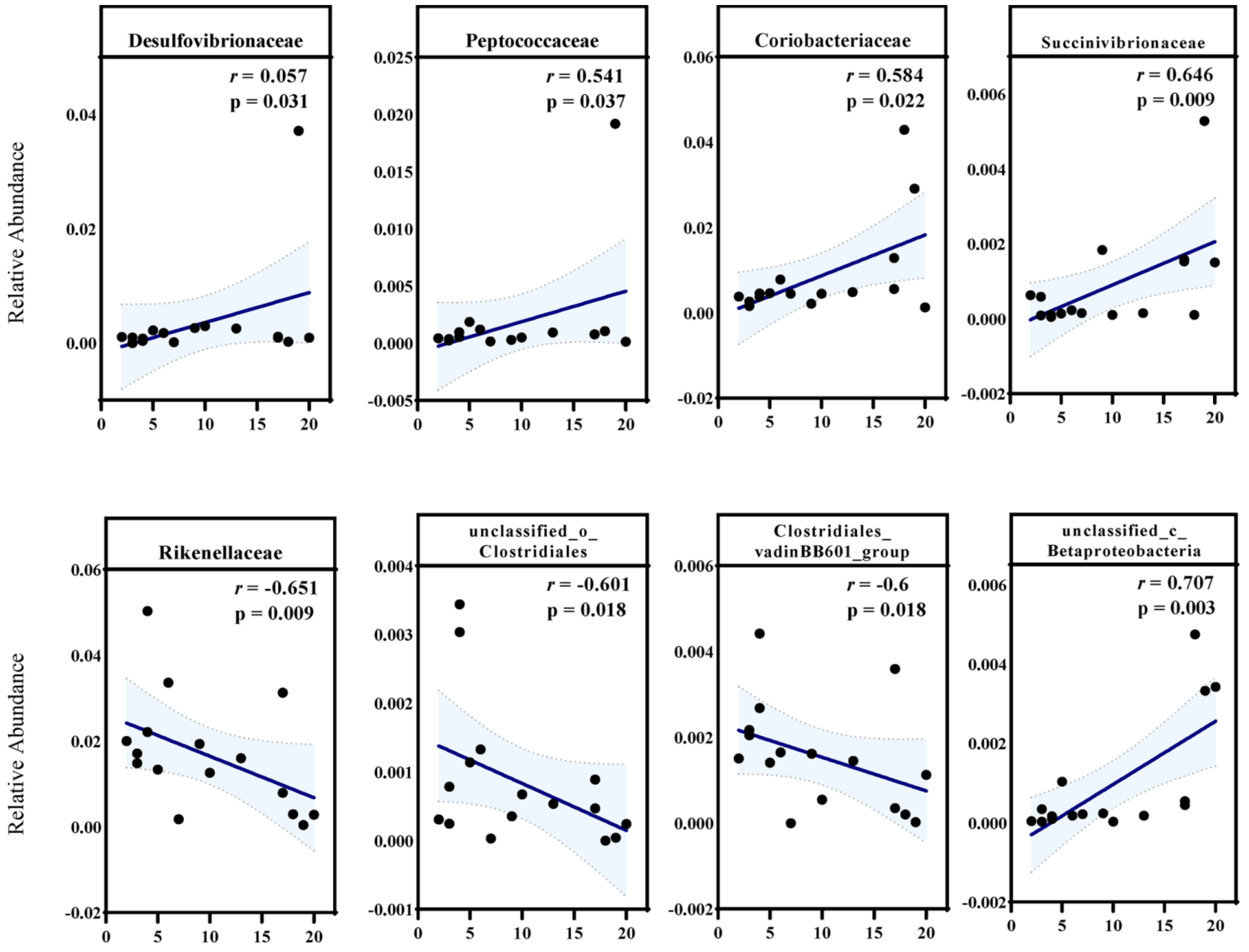

Supplementary Figure 3. The change trend of differential microbes at the family level related to age. The correlationship was tested by Pearson correlation analyses and adjusted by partial correlation analysis for excluding the confounders for controlling the False Discovery Rate (FDR).

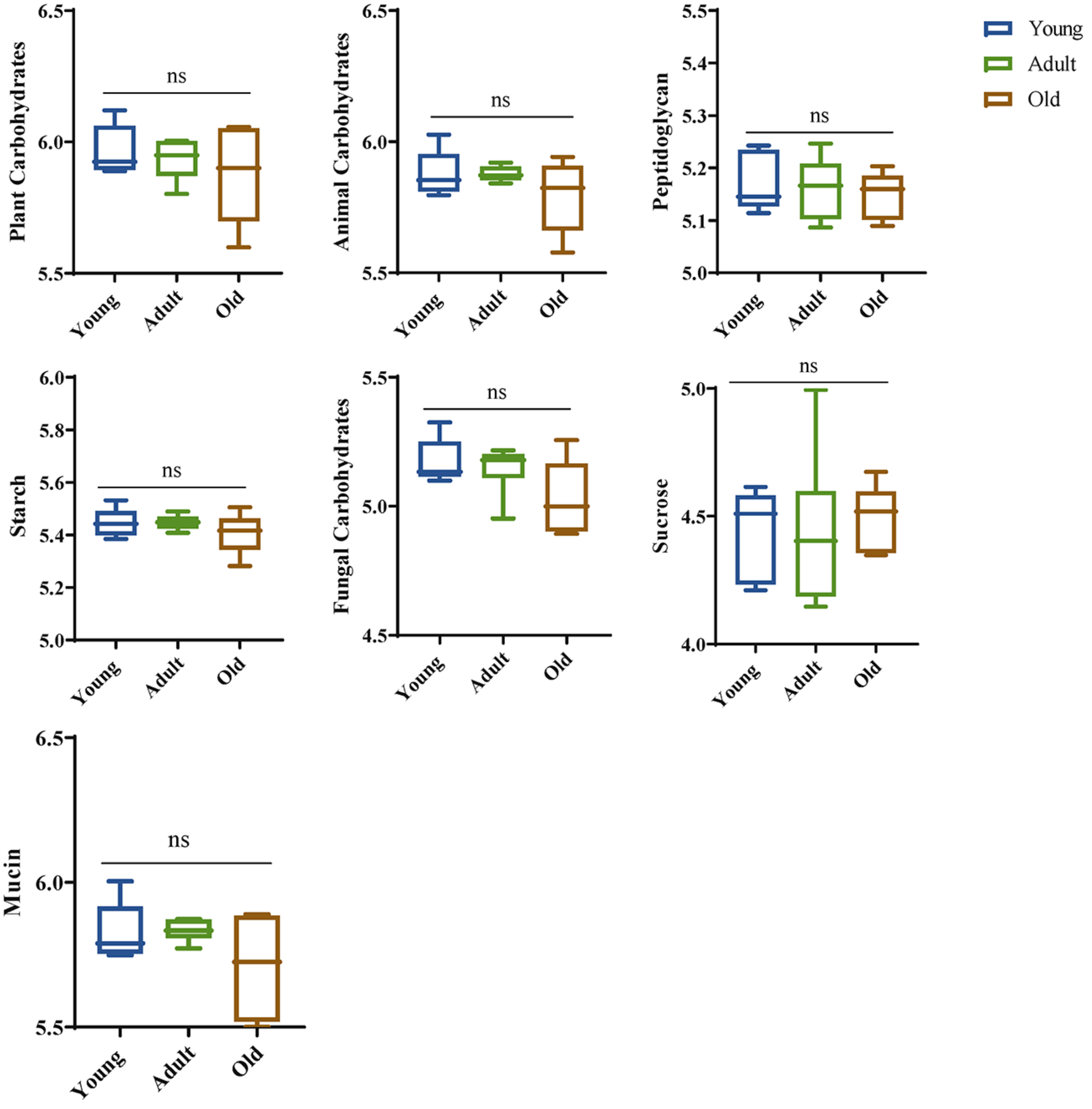

Supplementary Figure 4. Microbial functions in CAZymes among three groups. Representation of CAZymes in metagenomic data sets related to multiple classes of polysaccharides are plotted by their respective distributions.
