# Supplementary material for: Age-related changes in microbial composition and function in cynomolgus macaques
Source: Aging (Albany NY). 2019 Dec 14;11(24):12080–96. doi: 10.18632/aging.102541 (PMC6949106; doi:10.18632/aging.102541)
Supplement: Supplementary Table 1 [file aging-11-102541-s001..pdf]

SUPPLEMENTARY TABLE

Supplementary Table 1. The detail characteristics of the recruited monkeys.

characteristics	Young	Adult	Old	p-value
Sample size	5	6	5	-
Age (year)	3.2 ± 0.83	8.3 ± 2.94	18.2 ± 1.30	< 0.001
Age range	2~4	5~13	17~20	-
Sex(M/F)	Female	Female	Female	-
Weight (kg)	3.12 ± 0.85	3.99 ± 0.23	4.34 ± 0.67	0.023

p-value obtained from One-way analysis of variance(ANOVA). Characteristics are represented by mean ± SD.
